# Integration of longitudinal and circumferential strain predicts volumetric change across the cardiac cycle and differentiates patients along the heart failure continuum

**DOI:** 10.1186/s12968-023-00969-2

**Published:** 2023-10-02

**Authors:** T. Jake Samuel, Andrew P. Oneglia, Daisha J. Cipher, Justin A. Ezekowitz, Jason R. B. Dyck, Todd Anderson, Jonathan G. Howlett, D. Ian Paterson, Richard B. Thompson, Michael D. Nelson

**Affiliations:** 1 College of Nursing and Health Innovation, University of Texas at Arlington, Arlington, TX USA; 2 Division of Cardiology, Department of Medicine, Johns Hopkins University School of Medicine, Baltimore, MD USA; 3 Mazankowski Alberta Heart Institute, University of Alberta, Edmonton, AB Canada; 4 Department of Medicine, University of Alberta, Edmonton, AB Canada; 5 Department of Pediatrics, University of Alberta, Edmonton, AB Canada; 6 Libin Cardiovascular Institute of Alberta, Calgary, AB Canada; 7 Department of Cardiac Sciences, University of Calgary, Calgary, AB Canada; 8 University of Ottawa Heart Institute, University of Ottawa, Ottawa, ON Canada; 9 Department of Biomedical Engineering, University of Alberta, Edmonton, AB Canada; 10 Applied Physiology and Advanced Imaging Laboratory, Department of Kinesiology, University of Texas at Arlington, 676 W. Nedderman Dr., Arlington, TX 76019 USA

**Keywords:** Strain, Volume-time, Ejection fraction, Filling rate, Ejection rate

## Abstract

**Background:**

Left ventricular (LV) circumferential and longitudinal strain provide important insight into LV mechanics and function, each contributing to volumetric changes throughout the cardiac cycle. We sought to explore this strain-volume relationship in more detail, by mathematically integrating circumferential and longitudinal strain and strain rate to predict LV volume and volumetric rates of change.

**Methods:**

Cardiac magnetic resonance (CMR) imaging from 229 participants from the *Alberta HEART Study* (46 healthy controls, 77 individuals at risk for developing heart failure [HF], 70 patients with diagnosed HF with preserved ejection fraction [HFpEF], and 36 patients with diagnosed HF with reduced ejection fraction [HFrEF]) were evaluated. LV volume was assessed by the method of disks and strain/strain rate were assessed by CMR feature tracking.

**Results:**

Integrating endocardial circumferential and longitudinal strain provided a close approximation of LV ejection fraction (EF_Strain_), when compared to gold-standard volumetric assessment (EF_Volume_: *r* = 0.94, *P* < 0.0001). Likewise, integrating circumferential and longitudinal strain rate provided a close approximation of peak ejection and peak filling rates (PER_Strain_ and PFR_Strain_, respectively) compared to their gold-standard volume-time equivalents (PER_Volume_, *r* = 0.73, *P* < 0.0001 and PFR_Volume_, *r* = 0.78, *P* < 0.0001, respectively). Moreover, each integrated strain measure differentiated patients across the HF continuum (all *P* < 0.01), with the HFrEF group having worse EF_Strain_, PER_Strain_, and PFR_Strain_ compared to all other groups, and HFpEF having less favorable EF_Strain_ and PFR_Strain_ compared to both at-risk and control groups.

**Conclusions:**

The data herein establish the theoretical framework for integrating discrete strain components into volumetric measurements across the cardiac cycle, and highlight the potential benefit of this approach for differentiating patients along the heart failure continuum.

## Background

The importance of evaluating left ventricular (LV) strain in clinical imaging studies of patients across the heart failure (HF) continuum is well established [1–3], with strain often outperforming volumetric measures, such as ejection fraction, for risk prediction [4–6]. However, the integrated effects of strain ultimately determine volumetric function and thus it is important to understand the relationship between the distinct strain components and volume changes. Due to the unique muscle fiber orientation of the LV, tissue deformation occurs in well characterized complex patterns [7–9] which results in the shortening and lengthening of the myocardial borders in the circumferential and longitudinal direction (i.e. circumferential and longitudinal strain) [9–12]. Circumferential and longitudinal strains on the endocardial surface, in particular, determine the changes in LV volume across the cardiac cycle [13–16]. Indeed, changes in volume of a prolate ellipsoid (the shape of LV) are linearly related to changes in chamber length and quadratically related to changes in chamber circumference [15–17]. This strain-volume relationship helps to explain how ejection fraction can be maintained despite abnormalities in systolic strain for one component [15, 18], and informs the relative contributions of each strain component to volumetric function.

Despite a general appreciation for this strain-volume relationship, this concept has yet to translate to clinical populations or to relate systolic and diastolic strain rates to volumetric patterns of ejection and filling, respectively. As such, we leveraged a database of cardiac magnetic resonance (CMR) imaging from individuals along the HF continuum enrolled from the University of Alberta site of the *Alberta Heart Study* [19] to test the hypothesis that measures of systolic and diastolic function (LV ejection fraction, peak filling rate and peak ejection rate) derived from circumferential and longitudinal strain (a) correlate to gold-standard volume-time relationships, and (b) differentiate patients along the heart failure continuum.

## Methods

### Study population

The *Alberta Heart Study* (NCT02052804)[19] was approved by the Health Research Ethics Boards at the University of Alberta, University of Calgary, and Covenant Health, and written informed consent was obtained prior to data collection. This sub-study of the larger clinical trial only included individuals enrolled from the University of Alberta site of the larger trial so that study procedures were uniformly performed on the same CMR scanner with the same acquisition parameters. Patients were sub-divided into four groups (Table 1): (1) Healthy control participants with no evidence of coronary artery disease, hypertension, diabetes mellitus, organ disease or replacement therapies; inflammatory or autoimmune conditions, and no history of cardiac medications. (2) Participants at risk for the development of HF, with either hypertension (defined as ≥ 3 medications or LV hypertrophy as evidenced by an electrocardiogram or by elevated gender-matched LV mass index on an imaging test), and/or history of diabetes and > 45 years of age, and/or presence of obesity (defined as body mass index > 30 kg/m^2^). Exclusion criteria included signs and symptoms of HF (i.e. dyspnea or fatigue) and known prior HF. (3) Patients with clinically diagnosed HF with preserved ejection fraction (HFpEF) with a LV ejection fraction > 45%. (4) Patients with clinically diagnosed HF with reduced ejection fraction (HFrEF) and a LV ejection fraction < 45%.

**Table 1:** Patient characteristics used for the validation of the integrated strain approach to volume-time relationships

	n = 79
Group contributions	
Healthy Control, n (%)	19 (24)
At-Risk, n (%)	23 (29)
HFpEF, n (%)	23 (29)
HFrEF, n (%)	14 (18)
Demographics	
Age, years	66 ± 12
Height, cm	168 ± 10
Weight, kg	78.2 ± 14.8
Female Sex, n (%)	46 (58)
Medical history	
Hypertension, n (%)	47 (59)
Diabetes, n (%)	20 (25)
Smoking history, n (%)	33 (42)
Atrial fibrillation or flutter, n (%)	15 (19)
COPD, n (%)	6 (8)
Medications	
ARB or ACEi, n (%)	50 (63)
β-blocker, n (%)	39 (49)
Statins, n (%)	39 (49)
Antiplatelet, n (%)	2 (3)
Biochemistry	
NT-proBNP, pmol/L	18 (7–61)
Creatinine, µmol/L	85 (72–104)
Left ventricular morphology and function	
LV mass, g	116 (84–159)
LV mass index, g/m^2^	58 (48–78)
EDV, mL	146 (115–205)
EDV index, mL/m^2^	77 (64–105)
ESV, mL	63 (42–101)
ESV index, mL/m^2^	32 (24–53)
Stroke volume, mL	75 (64–97)
Stroke index, mL/m^2^	40 (35–50)
Ejection Fraction, %	59 (45–64)
Concentricity index, g/mL	0.76 (0.66–0.86)

Data are counts and percentages, mean ± standard deviation or median and interquartile range. *HFpEF* heart failure with preserved ejection fraction, *HFrEF* heart failure with reduced ejection fraction, *COPD* chronic obstructive pulmonary disease, *ARB* angiotensin receptor blocker, *ACEi* angiotensin converting enzyme inhibitor, *NT-proBNP* N-terminal pro brain natriuretic peptide, *LV* left ventricular, *EDV* end-diastolic volume, *ESV* end-systolic volume

### CMR protocol

All CMR examinations were performed utilizing a 1.5T clinical MRI system (Sonata; Siemens Medical Solutions, Erlangen, Germany), and all image acquisitions were retrospectively gated using an electrocardiogram and performed during breath-holds at end-expiration. LV morphology and function were measured from a series of short-axis balanced steady-state free precession cine images spanning the entire LV, along with two- and four-chamber long-axis images. Typical imaging parameters were: slice thickness of 8 mm with 2 mm gap between slices, echo time of 1.3 ms, repetition time of 2.6 ms, flip angle of 51°, field of view of 300×400 mm, and matrix size of 144 × 256, 930 Hz/pixel bandwidth, rate 2 GRAPPA parallel imaging and 10–14 views per segment reconstructed to 30 phases over the cardiac cycle for an acquired temporal resolution of 29–40 ms.

End-diastolic and end-systolic LV volumes and mass were measured using commercially available image analysis software, Syngo Argus, (Siemens Healthineers) by an experienced CMR interpreter (I.P.). In a subset of individuals (n = 79), LV volume-time relationships were determined, using commercially available software (version 5.6.8 cvi^42^; Circle Cardiovascular Imaging Inc., Calgary, Alberta, Canada), by manually delineating the endocardial borders of each short-axis slice at end-diastole, and during each cardiac phase between end-systole and diastasis by a single blinded interpreter (T.J.S.), as previously described [20]. The LV basal and apical boundaries were identified using the long-axis views to further define the extent of the LV chamber. Papillary muscles and trabeculae were included as part of the ventricular lumen. LV volumes were calculated by the summation of the volumes for each short-axis slice. PER_Volume_ and PFR_Volume_ were defined as the maximal LV volumetric change between sequential temporal phases, normalized to the LV end-diastolic volume [20].

### Endocardial strain and strain rate

Global endocardial LV strain/strain rate were assessed using commercially available software (version 5.13 cvi^42^; Circle Cardiovascular Imaging Inc., Calgary, Alberta, Canada), modified by the developers to export strain values derived from the endocardial border. Briefly, the endocardial and epicardial borders of the LV were manually traced at end-diastole and end-systole using a series of short-axis cines spanning the LV from base to apex, along with a horizonal long axis image (4-chamber) and vertical long axis image (2-chamber). The most basal short-axis slices that included LV outflow tract and the most apical slices without clear delineation of the luminal border were excluded from the analysis. Following this, the feature tracking algorithm was applied, and quality of tracking was confirmed throughout the whole cardiac cycle. Feature tracking analysis was performed by a single experienced interpreter (M.D.N), blinded to the clinical condition of each participant. Intra-observer reliability, expressed as the mean ± SD of the coefficient of variation, are as follows: circumferential strain, 1.3 ± 1.0%; systolic circumferential strain rate, 4.9 ± 7.4%; early diastolic circumferential strain rate, 3.4 ± 2.5%; late diastolic circumferential strain rate, 3.9 ± 3.8; longitudinal strain, 4.1 ± 4.2%; systolic longitudinal strain rate, 6.7 ± 7.6%; and early diastolic longitudinal strain rate, 6.4 ± 3.9%. Interobserver reliability, expressed as the mean ± SD of the coefficient of variation, are as follows: circumferential strain, 4.8 ± 5.0%; systolic circumferential strain rate, 6.9 ± 7.7%; early diastolic circumferential strain rate, 4.4 ± 7.1%; late diastolic circumferential strain rate, 4.6 ± 3.2; longitudinal strain, 4.7% ± 5.7%; systolic longitudinal strain rate, 7.0 ± 6.0%; and early diastolic longitudinal strain rate, 8.5 ± 7.7%.

### Integrated strain and strain rate

We used the integration of both circumferential and longitudinal endocardial strain and strain rate to calculate LV ejection fraction (EF_Strain_), the peak ejection rate (PER_Strain_), and peak filling rate (PFR_Strain_) based on the following theoretical framework:

1 $$V=k\cdot {C}^{2}\cdot L,$$

where V is LV volume, k is a shape constant, C is the basal short-axis circumference, and L is LV length. This approximate volume is used here only to represent the linear and quadratic contributions of length and circumference to volume, respectively. Based on Eq. 1, LV EF can be calculated by,

2 $$EF= \frac{\left(EDV-ESV\right)}{EDV} = \left(1-\frac{ESV}{EDV}\right) = 1-\frac{k\cdot {{C}_{ESV}}^{2}\cdot {L}_{ESV}}{k\cdot {{C}_{EDV}}^{2}\cdot {L}_{EDV}}$$

where EDV and ESV are the LV end-diastolic and end-systolic volume, respectively. As, peak longitudinal and circumferential strain (LS and CS, respectively) are calculated as:

3 $$LS= \frac{\left({L}_{1}-{L}_{0}\right)}{{L}_{0}} = \frac{\left({L}_{EDV}-{L}_{ESV}\right)}{{L}_{EDV}}, LS=1-\frac{{L}_{ESV}}{{L}_{EDV}}$$

4 $$CS= \frac{\left({C}_{1}-{C}_{0}\right)}{{C}_{0}} = \frac{\left({C}_{EDV}-{C}_{ESV}\right)}{{C}_{EDV}}, CS=1-\frac{{C}_{ESV}}{{C}_{EDV}}$$

where, L_0_ and C_0_ represent lengths at end-diastole and L_1_ and C_1_ represent lengths at end-systole, you can solve for L_ESV_ and C_ESV_ by the following:

5 $$\frac{{L}_{ESV}}{{L}_{EDV}}= 1-LS, or {L}_{ESV}= {L}_{EDV}\left(1-LS\right)\,and\,{C}_{EDV}\left(1-CS\right)$$

Substituting these formulas into Eq. 2 gives,

6 $$EF = 1 - \left[ {\frac{{C_{{EDV}} ^{2} \cdot \left( {1 - CS} \right)^{2} }}{{C_{{EDV}} ^{2} }}} \right]{\text{ }} \times \left[ {\frac{{L_{{EDV}} \cdot \left( {1 - LS} \right)}}{{L_{{EDV}} }}} \right] = 1 - \left( {1 - CS} \right)^{2} \times (1 - LS)$$

which is simplified to,

7 $$EF= \left[1-\left(1-LS\right)\times {\left(1-CS\right)}^{2}\right] \times 100$$

where LS and CS represent peak longitudinal strain (average of the 2- and 4-chamber long axis images) and peak circumferential strain (average of the two most basal LV slices that do not contain LV outflow tract), expressed as absolute decimal values, respectively. Briefly, Eq. 7 links the commonly measured fractional changes in linear dimensions of the heart (length and circumference) to corresponding fractional change in volume, which is the ejection fraction.

EF is the volumetric analog to peak systolic strain, and like strain has a value throughout the cardiac cycle, EF(t), and similarly the time rate of change of EF(t) is akin to strain rate, at any time in the cardiac cycle, based on the rate of change of the ventricular volume.

8 $$EF (t)= \frac{V (t) - {V}_{0}}{V_0}, \frac{dEF (t)}{dt}= {\frac{dV(t)}{dt}} \left/\vphantom{\frac{dV(t)}{dt}} V_{0} \right. .$$

V(t) is LV volume at any point in time, V_0_ is the initial (end-diastolic) volume, dEF(t)/dt is the time derivative of EF(t) at a given point in time, and dV(t)/dt is the time derivative of volume at a given time. Equation 8 shows that dEF(t)/dt is the rate of change of volume (i.e. ejection rate during systole and filling rate during diastole), normalized to peak volume (end-diastolic volume). Normalization of filling rates by V_0_ in this representation corrects for the effects of heart size on filling rates, and maintains the units of strain rate (/s) [21].

LV strain and strain rate can be related to the normalized filling rate dEF(t)/dt using the volume model introduced above:

9 $${V}^{\prime}=2\cdot k\cdot C\cdot L\cdot {C}^{\prime}+k\cdot {C}^{2}\cdot {L}^{\prime} \left(from\,the\,chain\,rule\right)\,and\,{V}_{0}=k{\cdot C}_{0}^{2}\cdot {L}_{0}.$$

Using the Lagrangian strain relationship in Eqs. 3 and 4 one can write,

10 $${C}_{0}= C \left/(CS+1) \right., {L}_{0}=L \left/ (LS+1 ) \right. \ and \ {C}^{\prime}=CSR_{{C}_{0}}, {L}^{\prime}=LSR_{{L}_{0}}.$$

where CSR is the circumferential strain rate derived using all available short-axis slices and LSR is longitudinal strain rate.

Substituting Eqs. 9 and 10 in Eq. 8, volumetric rates of change can be calculated as:

11 $$\frac{{dEF(T)}}{{dt}} = 2 \cdot CSR(t) \cdot (CS(t) + 1) \cdot (LS(t) + 1) + LSR(t) \cdot (CS(t) + 1)^{2}$$

and thus, the normalized PER_Strain_ and PFR_Strain_ can be estimated from longitudinal and circumferential strains and strain rates. Similar to Eq. 7 above, Eq. 11 links the commonly measured fractional changes in linear dimensions of the heart (length and circumference) and their rates of change (i.e. strain rates) to the corresponding rates of volume change. Importantly, dEF(t)/dt in Eq. 11, like the strains and strain rates it is derived from, is a normalized measure of volumetric function that is independent of the heart volume, with units of (s^−1^), similar to strain rates. PER_Strain_ was determined as the peak rate of blood ejection during systole and PFR_Strain_ was determined as the peak rate of volumetric filling in early diastole, equivalent to the s’ and e’ on the integrated strain curve, respectively.

### Statistical analysis

All statistical analyses were performed using SPSS (version 25, IBM SPSS Statistics, Armonk, NY) and GraphPad Prism (version 9.3.1, GraphPad Software, San Diego, CA). Our first hypothesis was that EF_Strain_, PER_Strain_ and PFR_Strain_ correlate to gold standard EF_Volume_, PER_Volume_ and PFR_Volume_. We tested this, in a sub-set of randomly selected participants (*n* = 79), using Pearson’s or Spearman’s correlation, as appropriate, and Bland–Altman plots.

To test our second hypothesis that EF_Strain_, PER_Strain_ and PFR_Strain_ could differentiate between patients along the HF continuum, group differences were assessed across the entire cohort of individuals (*n* = 229) by one-way analysis of variance or the Kruskal–Wallis test, as appropriate. Tukey’s and Dunn’s post-hoc corrections were performed when significant group main effects were observed in normally and non-normally distributed variables, respectively.

Categorical data were assessed by the Pearson’s chi-squared test after adjusting for multiple comparisons and presented as counts and percentages. Normal distribution and homoscedasticity were assessed with the Shapiro–Wilk test. Continuous data are presented as means ± standard deviation when normally distributed and median and interquartile range when not. The study alpha was set to α = 0.05.

## Results

Participant characteristics for the validation cohort are shown in Table 1.

**Fig. 1:**
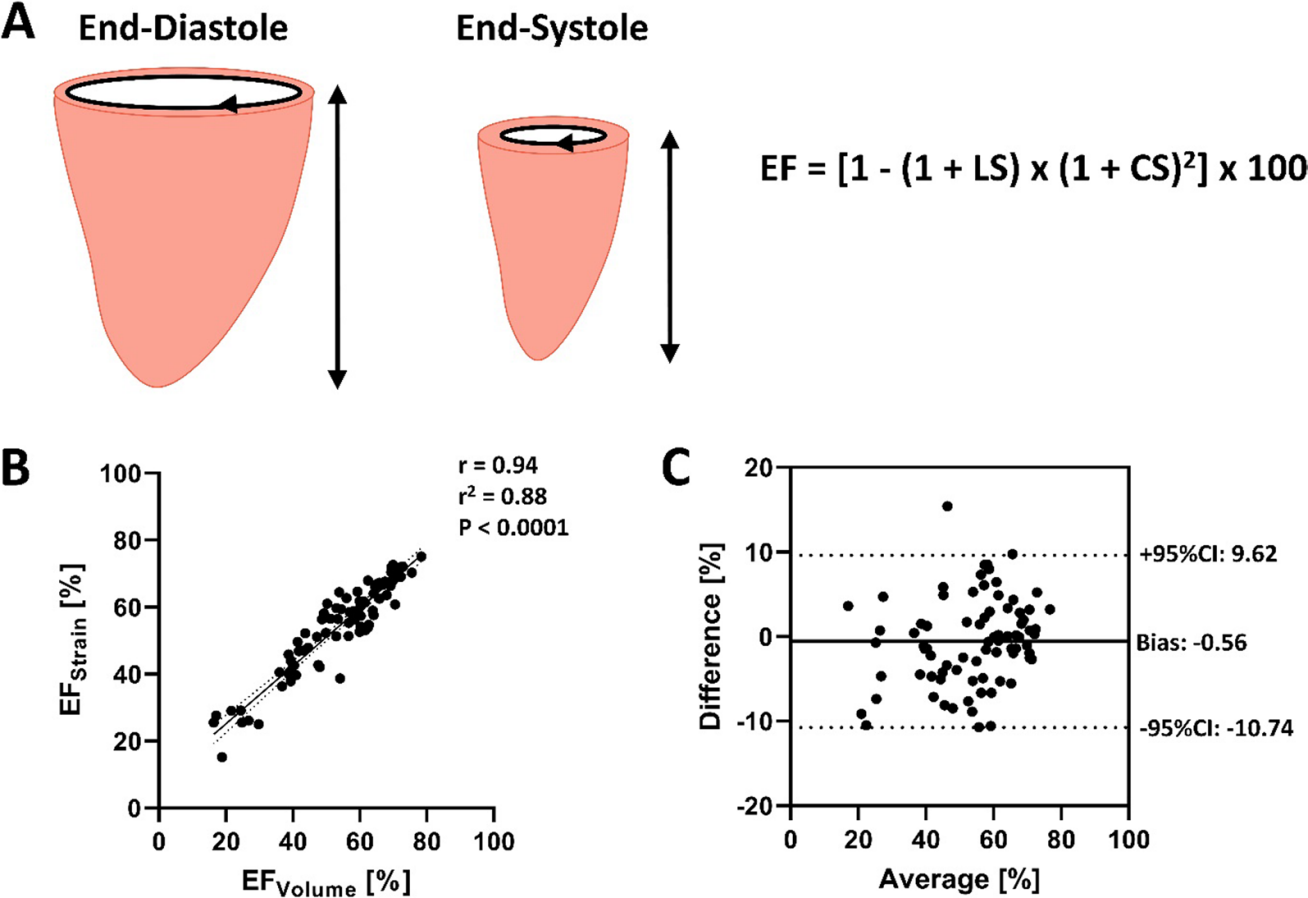
Left ventricular ejection fraction (EF) can be calculated by integrating peak longitudinal (LS) and circumferential strain (CS), with changes in ventricular volume being linearly related to changes in chamber length and quadratically related to changes in chamber circumference (**A**).[15] In a randomly chosen sample of 79 individuals with varying EF and clinical status, EF calculated using the integrated strain approach (EF_Strain_) and EF measured using the gold-standard method of disks volume-time relationship (EF_Volume_) were strongly related (**B**), with good agreement between the two measures across a range of EF (**C**)

**Fig. 2:**
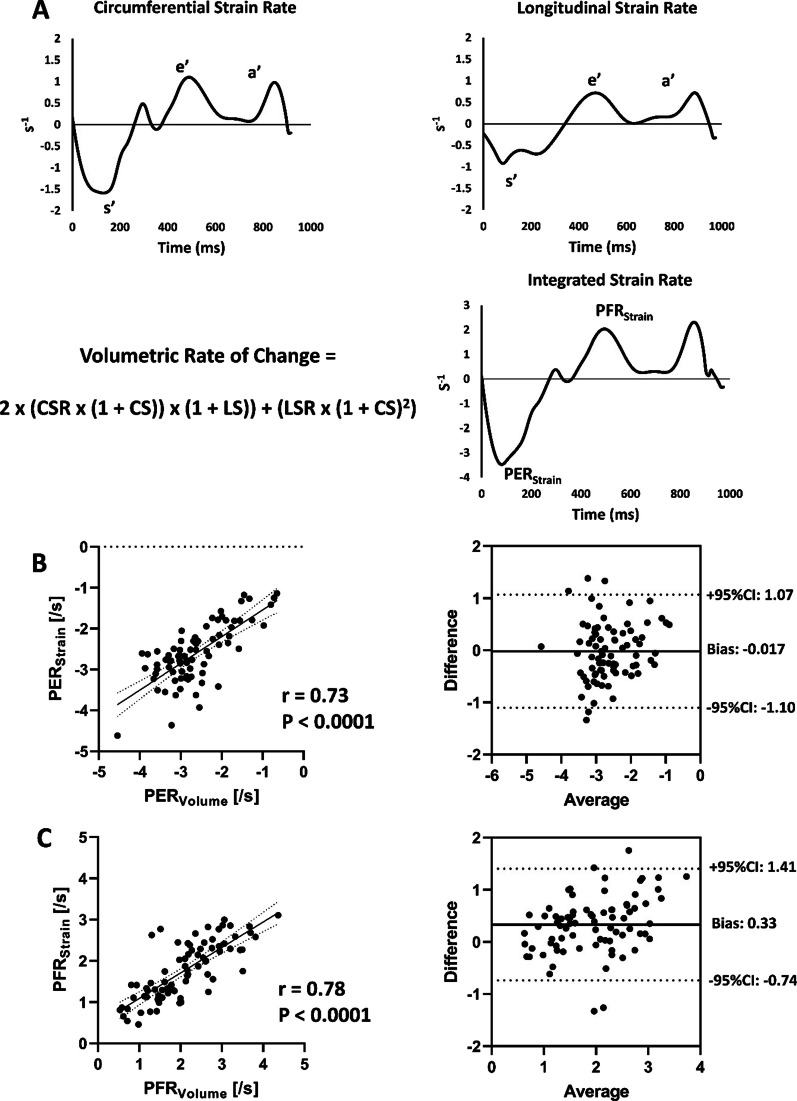
Data from a representative individual showing the circumferential and longitudinal strain rate curves as well as how using the integrated strain approach can be used to calculate the volumetric rate of change curve (**A**). From the integrated strain curve, peak ejection rate (PER_Strain_) and peak filling rate (PFR_Strain_) can be identified. PER_Strain_, calculated using the integrated strain approach and peak ejection rate measured using the gold-standard volume-time relationship (PER_Volume_) was correlated with good agreement between the two measures across a range of ventricular systolic performance (**B**). Similarly, PFR_Strain_ calculated using the integrated strain approach and peak filling rate measured using the gold-standard volume-time relationship (PFR_Volume_) was correlated with good agreement between the two measures in participants with varying degrees of diastolic dysfunction (**C**). These data were generated using the same randomly chosen sample of 79 individuals as in Fig. 1. s’ – peak systolic strain rate; e’ – peak early diastolic strain rate; a’ – peak late diastolic strain rate

EF_Strain_ was closely related to EF_Volume_ (*r* = 0.94, *P* < 0.0001; Fig. 1), with a bias of -0.56% (95%CI: -10.7 to 9.6%). Likewise, PER_Strain_ and PFR_Strain_ were moderately related to their volume-time equivalents PER_Volume_ and PFR_Volume_ (*r* = 0.73, *P* < 0.0001 and *r* = 0.78, *P* < 0.0001, Fig. 2), with a bias of − 0.017 s^−1^ (95%CI: − 1.10 to 1.07 s^−1^) and 0.33 s^−1^ (95%CI: − 0.74 to 1.41 s^−1^), respectively.

**Fig. 3:**
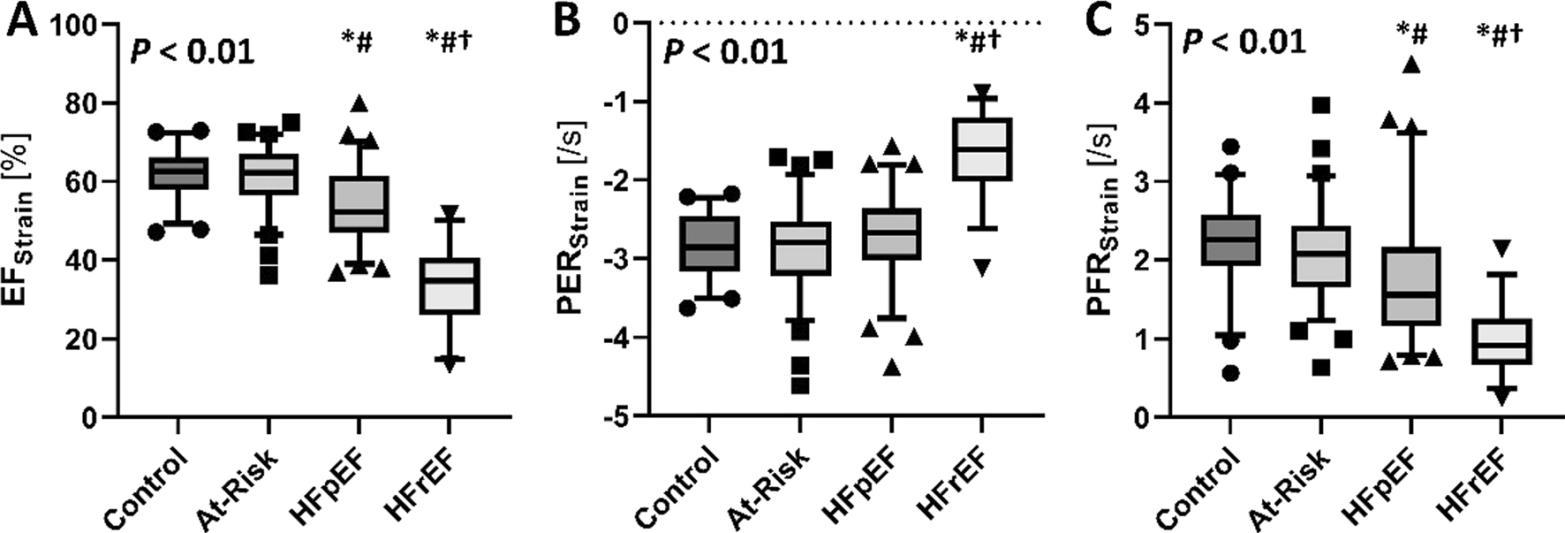
When applied to the entire cohort of subjects (n = 229), the integrated strain approach for measuring left ventricular ejection fraction (EF_Strain_), peak ejection rate (PER_Strain_) and peak filling rate (PFR_Strain_) successfully differentiated groups according to their heart failure diagnosis (all *P* < 0.01, Panels **A–C**). *indicates significantly different from control; ^#^indicates significantly different from At-Risk. ^†^indicates significantly different from HFpEF

The integrated strain approach was then applied across the entire cohort, with the patient characteristics, LV morphology and individual strain components for each of the groups found in Tables 2 and 3. EF_Strain_, PER_Strain_, and PFR_Strain_ successfully differentiated patients along the HF continuum (Fig. 3A–C), with HFrEF patients demonstrating worse EF_Strain_, PER_Strain_, and PFR_Strain_ than all other groups, and HFpEF having less favorable EF_Strain_ and PFR_Strain_ compared to both At-Risk and controls.

**Table 2:** Patient characteristics for the entire cohort (*n* = 229)

Variable	Healthy control *n* = 46	At-risk *n* = 77	HFpEF *n* = 70	HFrEF *n* = 36	Group effect
Demographics					
Age, years	65 (54–72)	65 (59–72)	70 (63–78)*	65 (58–76)	**0.026**
Height, cm	168 (160–173)	168 (161–178)	168 (163–175)	173 (166–179)	0.11
Weight, kg	72 (61–81)	78 (67–86)	86 (76–99)*^#^	86 (76–97)*	** < 0.001**
BSA, m^2^	1.82 ± 0.17	1.90 ± 0.24	2.01 ± 0.22*^#^	2.03 ± 0.24*^#^	** < 0.001**
Female Sex, n (%)	33 (72)	44 (57)	34 (49)	13 (36)	**0.009**
Medical History					
Hypertension, n (%)	0 (0)	61 (79)	50 (71)	21 (58)	** < 0.001**
Diabetes, n (%)	0 (0)	14 (18)	27 (39)	12 (33)	** < 0.001**
Smoking history, n (%)	6 (13)	42 (55)	42 (60)	20 (56)	** < 0.001**
Atrial fibrillation or flutter, n (%)	0 (0)	14 (18)	26 (37)	12 (33)	** < 0.001**
COPD, n (%)	0 (0)	4 (5)	13 (19)	8 (22)	** < 0.001**
Medications					
ARB or ACEi, n (%)	0 (0)	53 (69)	57 (81)	32 (89)	** < 0.001**
β-blocker, n (%)	0 (0)	24 (31)	53 (76)	33 (92)	** < 0.001**
Statins, n (%)	1 (2)	38 (49)	47 (67)	21 (58)	** < 0.001**
Diuretics, n (%)	0 (0)	31 (40)	54 (77)	32 (89)	** < 0.001**
Antiplatelet, n (%)	0 (0)	3 (4)	5 (7)	4 (11)	0.12
Biochemistry					
NT-proBNP, pmol/L	7.1 (4.4–11.2)	7.0 (4.0–15.5)	39.5 (19.0–94.5)*^#^	101.0 (40.0–269.6)*^#^	** < 0.001**
Creatinine, µmol/L	74 (67–84)	78 (68–89)	97 (75–120)*^#^	88 (77–108)*^#^	** < 0.001**

Data are counts and percentages, mean ± standard deviation or median and interquartile range. *HFpEF* heart failure with preserved ejection fraction, *HFrEF* heart failure with reduced ejection fraction, *BSA* body surface area, *COPD* chronic obstructive pulmonary disease, *ARB* angiotensin receptor blocker, *ACEi* angiotensin converting enzyme inhibitor, *BP* blood pressure, *BNP* brain natriuretic peptide, *NT-proBNP* N-terminal pro brain natriuretic peptide; *indicates significantly different from control; ^#^indicates significantly different from At-Risk. Bold values highlight where there is a significant group effect

**Table 3:** Left ventricular morphology and function across the heart failure continuum

Variable	Healthy control *n* = 46	At-risk *n* = 77	HFpEF *n* = 70	HFrEF *n* = 36	Group effect
Cardiac morphology					
LV mass, g	88 (80–105)	107 (83–132)*	133 (116–159)*^#^	174 (141–204)*^#†^	** < 0.001**
LV mass index, g/m^2^	49 (45–55)	56 (49–69)	67 (60–77)*^#^	86 (73–100)*^#†^	** < 0.001**
LV EDV, mL	125 (115–146)	125 (108–165)	144 (122–179)	251 (178–331)*^#†^	** < 0.001**
LV EDV index, mL/m^2^	71 (63–78)	68 (61–84)	73 (62–98)	123 (94–156)*^#†^	** < 0.001**
LV ESV, mL	48 (37–57)	46 (37–66)	65 (48–89)*^#^	171 (119–241)*^#†^	** < 0.001**
LV ESV index, mL/m^2^	26 (21–30)	25 (20–31)	32 (25–47)*^#^	85 (61–114)*^#†^	** < 0.001**
LV SV, mL	79 (70–90)	82 (68–100)	82 (66–98)	76 (61–84)	0.13
LV stroke index, mL/m^2^	43 (40–48)	44 (37–50)	41 (34–48)	36 (29–41)*^#†^	** < 0.001**
LV ejection Fraction, %	62 (59–65)	63 (59–69)	54 (47–62)*^#^	31(25–37)*^#†^	** < 0.001**
LV concentricity index, g/mL	0.72 (0.66–0.79)	0.81 (0.72–0.89)*	0.87 (0.77–1.00)*	0.69 (0.59–0.84)^#†^	** < 0.001**
LA volume index, mL/m^2^	50 (41–59)	46 (37–63)	57 (43–80)^#^	70 (51–85)*^#^	** < 0.001**
Global Left ventricular endocardial strain and strain rate					
Circumferential strain, %	− 29.6 ± 3.9	− 30.2 ± 5.4	− 25.5 ± 6.7*^#^	− 12.6 ± 4.1*^#†^	** < 0.001**
Systolic circumferential SR, s^−1^	− 1.32 (− 1.46–− 1.13)	− 1.37 (− 1.50–− 1.16)	− 1.31 (− 1.52–− 1.04)	− 0.67 (− 0.91–− 0.47)*^#†^	** < 0.001**
Early diastolic circumferential SR, s^−1^	1.22 (0.98–1.44)	1.18 (0.91–1.48)	0.77 (0.60–1.15)*^#^	0.42 (0.29–0.50)*^#†^	** < 0.001**
Late diastolic circumferential SR, s^−1^	0.74 (0.58–1.05)	0.79 (0.66–1.01)	0.75 (0.55–1.05)	0.45 (0.29–0.59)*^#†^	** < 0.001**
Longitudinal strain, %	− 21.5 ± 3.3	− 19.7 ± 3.8	− 16.9 ± 4.1*^#^	− 10.2 ± 3.9*^#†^	** < 0.001**
Systolic longitudinal SR, s^−1^	− 0.90 (− 1.02–− 0.81)	− 0.87 (− 1.00–− 0.79)	− 0.75 (− 0.94–− 0.63)*^#^	− 0.49 (− 0.65–− 0.37)*^#†^	** < 0.001**
Early diastolic longitudinal SR, s^−1^	0.84 (0.67–1.01)	0.74 (0.53–0.93)	0.55 (0.42–0.76)*^#^	0.31 (0.17–0.39)*^#†^	** < 0.001**
Late diastolic longitudinal SR, s^−1^	0.79 (0.66–0.89)	0.76 (0.62–0.92)	0.58 (0.42–0.77)*^#^	0.46 (0.29–0.65)*^#^	** < 0.001**

Data are counts and percentages, mean ± standard deviation or median and interquartile range. *HFpEF* heart failure with preserved ejection fraction, *HFrEF* heart failure with reduced ejection fraction, *LV* left ventricular, *EDV* end-diastolic volume; *ESV* end-systolic volume, *SR* strain rate; *indicates significantly different from control; ^#^indicates significantly different from At-Risk. ^†^indicates significantly different from HFpEF. Bold values highlight where there is a significant group effect

## Discussion

Circumferential and longitudinal deformation of the LV occurs simultaneously in systole and diastole, with each component contributing to LV ejection and filling, respectively. Here, we extend previous literature examining this strain-volume relationship, by showing that LV ejection fraction, along with peak ejection and peak filling rates, can be accurately derived by integrating discrete strain components along the cardiac cycle. The utility of this approach is highlighted by demonstrating that each integrated component effectively differentiates participants along the HF continuum.

**Fig. 4:**
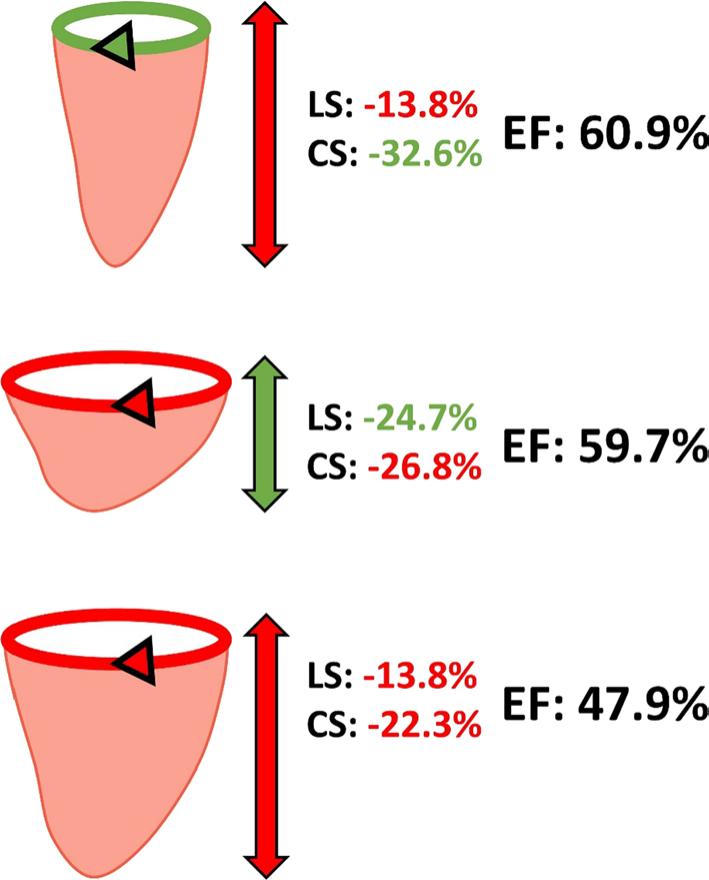
Three representative case examples are shown, highlighting the strain-volume relationship and the influence each individual strain component has on left ventricular ejection fraction (EF). *Top:* 63-year-old male with heart failure with preserved EF. *Middle:* 55-year-old female control participant. *Bottom:* 80-year-old female with heart failure with reduced EF. Note how a reduction in strain in one direction can be compensated for by a higher strain in the other direction to achieve the same EF, while reductions in both strain components ultimately leads to a reduction in EF. Together, these examples highlight the potential advantages of integrating discrete strain components when interpreting global left ventricular function. Green arrows and numbers represent normal strains, while red arrows and numbers represent impaired strains, relative to the mean of the control group (LS: − 21.5 ± 3.3%; CS: − 29.6 ± 3.9%; EF: 62.0 ± 6.2%)

Impaired LV strain is a well-established indicator of poor clinical status in a variety of populations [15, 18, 22–24]. However, interpretation of LV strain is often complicated when one principal strain component is elevated while the other is reduced [15, 18, 25, 26]. The potential major advantage of integrating circumferential and longitudinal strain to calculate LV ejection fraction, over conventional discrete strain approaches, is that both measures are condensed into a single measure of overall LV volumetric function. Indeed, LV ejection fraction is the product of circumferential and longitudinal tissue deformation, each contributing independently to volumetric changes across the cardiac cycle. This strain-volume relationship therefore helps to explain how ejection fraction can be maintained (or even increase) despite abnormalities in systolic strain in one direction [15, 18], providing valuable insight into the relative contributions of each strain component to volumetric function (as illustrated in Fig. 4).

The integration of circumferential and longitudinal strain rates to predict peak ejection and peak filling rates extends prior reports that have focused only on LV ejection fraction [13–16]. While the clinical utility of assessing peak ejection and peak filling rates is well established [20, 21, 27–33], the approach is typically dependent upon either invasive LV catheterization methods [21, 29] or on manual contouring of images of the LV to generate volume-time relationships [20, 27]. Indeed, invasive LV hemodynamic assessment is associated with high risk and is often not feasible in many clinical and sub-clinical populations. While non-invasive assessment of volumetric ejection and filling rates using image-based volume-time curves avoids many of these limitations, this approach is time-consuming and highly user-dependent, reducing its overall clinical utility and feasibility. Thus, the proposed integrated strain approach offers a major advantage, given that most strain analysis is semi-automated, with minimal user input. Integrating circumferential and longitudinal strain rates is also attractive because it has the potential to reduce the overall number of endpoint measurements reported and expresses the results in volume normalized units of measure.

*Experimental Considerations.* The theoretical framework for which the integrated strain concept is based is specific for endocardial strain and strain rates only. Indeed, due to the gradual change in fiber orientation and associated strain seen from the LV endocardial to mid-wall layers [7, 10–12], inclusion of information not exclusive to the endocardial border in-validates the mathematical assumptions of the current work. This is important, because strain is typically reported as a transmural strain. However, strain outputs denoted as “endocardial” often do not reflect strain from the endocardial border, but rather a region of myocardium from the endocardium-to-midwall (e.g. 33% of the inner LV layer). Future studies wishing to adopt this integrated strain approach should therefore be cognizant of this important distinction.

## Limitations

The sample size was relatively small, and it remains unknown whether integrating strain is superior for predicting clinical outcomes compared to traditional (discrete) global strain measures. However, these hypothesis generating results show that the integrated strain approach can successfully differentiate patients along the heart failure continuum, highlighting it’s clinical potential.

## Conclusions

With these considerations in mind, the data herein establish the theoretical framework for integrating discrete strain components into a single measure of LV ejection and filling rate. The data show that LV ejection fraction, along with peak ejection and peak filling rates, can be accurately derived by integrating discrete strain components along the cardiac cycle. The utility of this approach is highlighted by demonstrating that each integrated component effectively differentiates participants along the HF continuum.

## Data Availability

The datasets generated during and/or analyzed during the current study are not publicly available as data analysis remains ongoing but are available from the corresponding author on reasonable request.

## Abbreviations

CMR: Cardiac magnetic resonance
EF_Strain_: Ejection fraction from the integrated strain approach
EF_Volume_: Ejection fraction from the volume-time relationship
HF: Heart failure
HFpEF: Heart failure with preserved ejection fraction
HFrEF: Heart failure with reduced ejection fraction
LV: Left ventricular
PER_Strain_: Peak ejection rate from the integrated strain approach
PER_Volume_: Peak ejection rate from the volume-time relationship
PFR_Strain_: Peak filling rate from the integrated strain approach
PFR_Volume_: Peak filling rate from the volume-time relationship
